# Efficacy and safety of unilateral biportal endoscopy versus other spine surgery: A systematic review and meta-analysis

**DOI:** 10.3389/fsurg.2022.911914

**Published:** 2022-07-25

**Authors:** Bin Zheng, Shuai Xu, Chen Guo, Linyu Jin, Chenjun Liu, Haiying Liu

**Affiliations:** Spine Surgery Department, Peking University People's Hospital, Beijing, Beijing Municipality, China

**Keywords:** unilateral biportal endoscopic, percutaneous endoscopic lumbar discectomy (PELD), microendoscopic discectomy (MED), spine, minimally invasive surgery

## Abstract

**Background:**

This study aimed to evaluate the efficacy and safety of unilateral biportal endoscopy (UBE) versus other forms of spine surgery.

**Methods:**

Electronic databases were systematically searched up to February 2022. The authors used Review Manager 5.3 to manage the data and perform the review.

**Results:**

After the preliminary selection of 239 studies from electronic databases, the full inclusion criteria were applied; 16 studies were found to be eligible for inclusion. These 16 studies enrolled 1,488 patients: 653 patients in the UBE group, 570 in the microendoscopic discectomy group, 153 in the percutaneous endoscopic lumbar discectomy group, and 70 in the posterior lumbar interbody fusion group. UBE was superior to microendoscopic discectomy regarding 1-day Visual Analog Scale(VAS) back pain scores (*P* < 0.00001). No difference was found between UBE and microendoscopic discectomy regarding 1-day Visual Analog Scale leg pain scores (*P* = 0.25), long-term VAS back pain scores (*P* = 0.06), long-term VAS leg pain scores (*P* = 0.05), Oswestry Disability Index scores (*P* = 0.09) or complications (*P* = 0.19). Pooled analysis indicated that UBE was similar to percutaneous endoscopic lumbar discectomy regarding 1-day VAS back pain scores (*P* = 0.71), 1-day VAS leg pain scores (*P* = 0.37), long-term VAS back pain scores (*P* = 0.75), long-term VAS leg pain scores (*P* = 0.41), Oswestry Disability Index scores (*P* = 0.07) and complications (*P* = 0.88). One study reported no difference between UBE and posterior lumbar interbody fusion regarding long-term VAS back pain, long-term VAS leg pain, or Oswestry Disability Index scores.

**Conclusions:**

UBE is superior to microendoscopic discectomy to relieve back pain 1 day postoperatively. However, these two procedures are similar regarding 1-day leg pain relief, long-term effects, and safety. UBE and percutaneous endoscopic lumbar discectomy are similar regarding 1-day pain relief, long-term effects and safety. More evidence is needed to evaluate the efficacy and safety of UBE versus posterior lumbar interbody fusion.

## Introduction

The prevalence of spinal diseases has increased rapidly in all populations because of occupational ergonomic factors and extended life spans (1, 2). Among spinal disorders, lumbar spinal stenosis and lumbar disc herniation are the most common. Therefore, increasing attention is being focused on the search for appropriate treatment (3, 4). With the evolution of surgical techniques, minimally invasive spine surgery has emerged in recent decades as an alternative to conventional open surgery.

Unilateral biportal endoscopy (UBE) is a novel form of minimally invasive spine surgery. The roots of UBE date back to the 1980s, when Kambin tried to apply arthroscopy to lumbar discectomy (5). Unlike other endoscopic procedures that have evolved, spine endoscopy procedures that are currently mature—namely, percutaneous endoscopic lumbar discectomy (PELD) and microendoscopic discectomy (MED)—rely on one portal. By contrast, UBE involves two portals on one side. One portal is for optical instruments and the saline irrigation system; the other is for surgical instruments. UBE allows adequate decompression control and wider surgical vision than one-portal spine endoscopy, leading to satisfactory safety and effectiveness (6).

Compared with traditional open surgery, the minimally invasive surgical technique provides the benefits of less trauma, reduced pain, and faster healing. MED and PELD are two common and mature minimally invasive spinal surgeries. According to the surgical approach, PELD can be divided into percutaneous endoscopic interlaminar discectomy (PEID) and percutaneous transforaminal endoscopic discectomy (PTED). However, conventional open surgery remains the gold standard for degenerative spine disorders. UBE has been introduced as a novel treatment option for spine disease in recent years. Although this procedure has been used for a relatively short time, previous studies have reported satisfactory outcomes compared with baseline. As a novel technique, UBE requires comparison to other, more mature surgical procedures to verify its efficacy and safety.

The body of research on UBE is growing (6), but some comparisons between this procedure and other forms of spine surgery have been inconsistent. Meta-analysis is defined as first-level evidence, which is the highest level of evidence. High-quality systematic reviews and meta-analyses of UBE versus other forms of spine surgery are lacking. Therefore, we conducted this systematic review and meta-analysis of the published literature to evaluate the efficacy and safety of UBE versus other forms of spine surgery.

## Methods

The present systematic review and meta-analysis was performed according to the Preferred Reporting Items for Systematic Reviews and Meta-Analysis (PRISMA) guidelines.

### Search strategy

The authors searched PubMed, the Cochrane Library, Scopus, Embase, Ovid, and Web of Science up to February 2022 using the following search terms: “unilateral biportal endoscopy” or “biportal endoscopy spine surgery”. The reference lists of included articles were also screened to avoid missing relevant studies. The full text was obtained from Peking University Library.

### Inclusion criteria

The inclusion criteria, specified according to the PICOS framework, were as follows.

*P (patients):* Patients diagnosed with lumbar disc herniation or spinal stenosis.
*I (intervention):* Patients who underwent UBE surgery.
*C (comparison):* Patients who underwent other spine surgery, specifically PELD, MED or posterior lumbar interbody fusion (PLIF).
*O (outcomes):* The primary outcomes included 1-day VAS leg pain, 1-day VAS back pain, long-term VAS leg pain, long-term VAS back pain, and Oswestry Disability Index (ODI) scores. Complication events were included as secondary outcomes to evaluate the safety of UBE. To be included, studies were required to report at least one of the above outcomes.
*S (study design):* All comparative studies: case–control studies; cohort studies, randomized clinical trials. The included studies were required to report at least one of the above outcomes. Only studies published in peer-reviewed journals were considered for inclusion. Only literature published in English was eligible for inclusion.

### Exclusion criteria

I. Patients diagnosed with tuberculosis, infection, scoliosis, fractures, tumors or rheumatoid arthritis.
II. Studies or patients presented by other included articles; review studies; case reports; technical notes.
III. Cervical surgeries.

### Study selection and data extraction

Two authors independently screened the literature by reading the titles, abstracts, and full texts and applying the inclusion and exclusion criteria. The corresponding author, Liu, supervised the whole process and resolved all discrepancies.

Two authors worked independently to extract the following information: (1) general information: first author, publication year, and type of study; (2) participant characteristics: sample size, age, and baseline characteristics; (3) surgical procedure; (4) primary outcomes: 1-day VAS leg pain, 1-day VAS back pain, long-term VAS leg pain, long-term VAS back pain and ODI scores; and (5) secondary outcome: number of complications.

### Evaluation of risk of bias

Only randomized controlled trials (RCTs) were eligible for risk-of-bias assessment. Following the Preferred Reporting Items for Systematic Reviews and Meta-Analysis (PRISMA) and Cochrane Collaboration criteria, two authors independently assessed the risk of bias in the included studies in the following areas: (1) random sequence generation; (2) allocation concealment; (3) blinding of participants and personnel; (4) blinding of outcome assessment; (5) incomplete outcome data; (6) selective reporting; and (7) other bias. The Newcastle–Ottawa Scale was applied to evaluate observational studies.

### Data analysis

The authors performed the analysis using Review Manager 5.3. For continuous variables, mean differences (MDs) with 95% confidence intervals (CIs) were used to calculate pooled effects. For dichotomous outcomes, odds ratios (ORs) with 95% CIs were used to calculate pooled effects. Heterogeneity was represented using Higgins’ I-squared statistic (I^2^). A fixed- or random-effects model was used according to the degree of heterogeneity. A random-effects model was applied when heterogeneity was low (I^2^ > 50%); otherwise, a fixed-effects model was applied. Funnel plots were generated and examined to detect publication bias. Visual estimation was performed to examine the asymmetry of the funnel plot. The corresponding author supervised the whole process.

## Results

### Study selection

**Figure 1:**
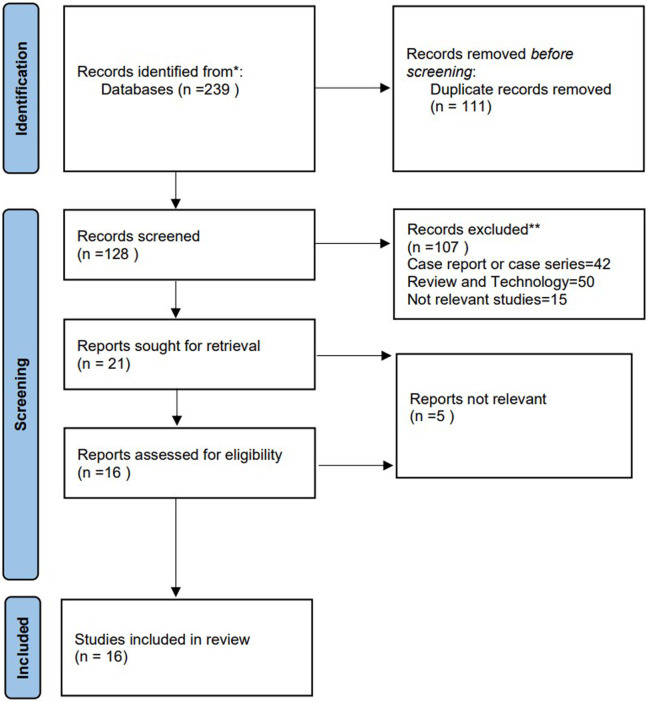
Flow chart of study selection.

The initial search included 239 studies. Excluding duplicates, 128 articles were screened by title and abstract. All 21 relevant articles were assessed: 16 studies met the inclusion criteria for data analysis. The study selection flow chart is shown in Figure 1. The 16 eligible articles (7–22) included 19 comparison groups, with a combined 653 patients who underwent UBE, 612 who underwent MED, 153 who underwent PELD, and 70 who underwent PLIF. Of the 16 included articles, 2 were RCTs, 5 were cohort studies, and 9 were case–control studies. In total, 1,488 patients were involved in this study. The baseline details are shown in Table 1.

**Table 1:** Included studies baseline.

Study	Study type	Surgical procedure		Sample Size		Vas back baseline	Vas leg baseline	ODI baseline	VAS back baseline	VAS leg baseline	ODI baseline
		EXP	CON	EXP	CON	EXP	EXP	EXP	CON	CON	CON
Choi 2018	Prospective Study	UBE	MED	20	20	6.52 ± 1.41	7.91 ± 1.47	31.70 ± 8.13	6.83 ± 1.95	7.81 ± 1.51	28.58 ± 11.67
			PEID		20				6.84 ± 2.05	8.25 ± 1.70	28.96 ± 9.41
			PTED		20				6.76 ± 1.61	7.67 ± 1.68	30.48 ± 9.23
Heo 2018	Case Control	UBE	MED	46	42	7.04 ± 1.38	7.96 ± 1.07	57.98 ± 5.83	6.76 ± 1.49	7.76 ± 1.08	57.98 ± 5.83
KIM 2018	Case Control	UBE	MED	60	81	6.22 ± 1.5	7.93 ± 1.0	70.15 ± 1.0	6.33 ± 1.5	7.98 ± 1.0	71.85 ± 8.4
Heo 2019	Prospective Study	UBE	MED	37	33	7.02 ± 1.34	8.05 ± 1.08	58.68 ± 5.57	6.64 ± 1.45	7.67 ± 1.08	56.36 ± 5.91
			PEID		27				7.04 ± 1.48	7.93 ± 1.07	56.70 ± 5.66
Heo 2019 II	Prospective Study	UBE	MED	23	36	non	8.1 ± 1.2	57.8 ± 6.3	/	7.7 ± 1.0	59.4 ± 5.9
Kang 2019	Randomized control trials	UBE	MED	32	30	6.3	/	/	6.2	/	/
Park 2019	Case Control	UBE	PLIF	71	70	6.0 ± 1.5	6.6 ± 1.3	61.9 ± 8.2	5.4 ± 2	7.0 ± 1.7	55.7 ± 12.1
MIN 2019	Case Control	UBE	MED	54	35	5.27 ± 0.91	7.38 ± 0.65	60.4 ± 6.88	5.34 ± 0.96	7.37 ± 0.94	61.1 ± 4.89
KIM 2020	Case Control	UBE	MED	32	55	6.2 ± 1.3	7.9 ± 0.6	68.1 ± 5.4	6.5 ± 1.5	7.8 ± 1.7	69.6 ± 6.2
Park 2020	Randomized control trials	UBE	MED	32	32	6.1 ± 2.6	6.5 ± 1.7	46.2 ± 20.5	6.1 ± 2.4	7.4 ± 2.1	47.0 ± 14.4
Aygun 2021	Prospective study	UBE	MED	77	77	/	/	53.18	/	/	51.39
Ito 2021	Case-control	UBE	MED	42	139	3.9 ± 1.2	3.9 ± 1.3	23.5 ± 9.2	3.8 ± 1.1	4.5 ± 1.2	23.3 ± 9.8
Kang 2021	Case Control	UBE	MED	47	32	/	/	/	/	/	/
Hao 2022	Case Control	UBE	PELD	20	20	7.23 ± 0.82	7.05 ± 0.59	71.52 ± 3.68	7.46 ± 0.77	7.20 ± 0.57	71.03 ± 4.79
Hua 2022	Case Control	UBE	PELD	36	36	5.4 ± 1.2	7.0 ± 0.9	51.4 ± 4.2	5.6 ± 1.4	7.2 ± 0.8	53.2 ± 4.5
Jiang 2022	Case Control	UBE	PELD	24	30	5.75 ± 0.99	7.04 ± 2.12	62.25 ± 13.57	6.00 ± 0.95	7.10 ± 1.56	64.20 ± 9.46

MED: MicroEndoscopic Discectomy.

PELD: Percutaneous Endoscopic Lumbar Discectomy.

PEID: Percutaneous Endoscopic Interlaminar Endoscopy.

PTED: Percutaneous Transforaminal Endoscopic Discectomy.

PLIF: Posterior Lumbar Interbody Fusion.

EXP: Experimental group.

CON: Control group.

### Risk of bias

**Figure 2:**
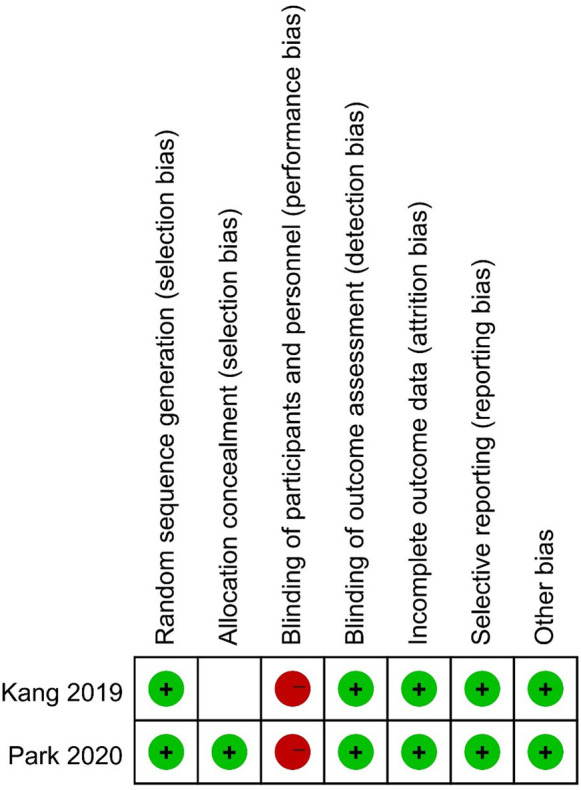
Risk of Bias of RCTs.

We used the Cochrane Collaboration tool to evaluate the quality of the 2 RCTs. None of the RCTs was double-blinded. Kang 2019 reported no details on allocation concealment. The details regarding the risk of bias in the two studies are shown in Figure 2. The results of the quality evaluation of the observational studies are listed in Table 2.

**Table 2:** Risk of bias assessment using the Newcastle–Ottawa Scale for observational studies.

Study	Selection			Comparability			Outcome			Total scores
	Exposed Cohort	Non-exposed Cohort	Ascertainment Of exposure	Outcome of interest	The most important factor	Additional factor	Assessment of outcomes	Length of follow up	Adequacy of follow up	
Choi 2018	★	★	★	★	★	★	★	⋆	⋆	7
Heo 2018	★	★	★	★	★	★	★	★	★	9
Kim 2018	★	★	★	★	★	★	⋆	★	★	8
Heo 2019	★	★	★	★	★	★	★	★	★	9
Heo 2019II	★	★	★	★	★	★	★	★	★	9
Kang 2019	★	★	★	★	⋆	★	★	★	★	8
Min 2019	★	★	★	★	★	★	★	★	★	9
Kim 2020	★	★	★	★	★	★	★	★	★	9
Park 2020	★	★	★	★	★	★	★	★	★	9
Aygun 2021	★	★	★	★	★	★	★	★	★	9
Ito 2021	★	★	★	★	★	★	⋆	★	★	8
Kang 2021	★	★	★	★	★	★	⋆	★	★	8
Hao 2022	★	★	★	★	★	★	★	★	★	9
Hua 2022	★	★	★	★	★	★	★	★	★	9
Jiang 2022	★	★	★	★	★	★	⋆	★	★	8

### UBE vs. MED

#### VAS back pain scores at 1 day

**Figure 3:**
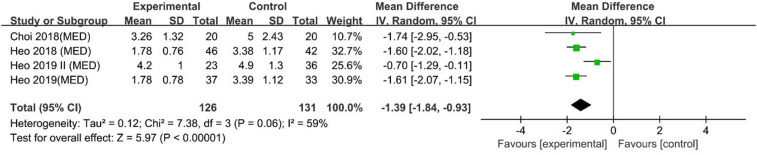
Forrest plot of vas back pain 1-day UBE vs MED.

A pooled analysis of 4 studies indicated that UBE was superior to MED for 1-day VAS back pain scores [MD: −1.39, 95% CI: (−1.84, −0.93), *P* < 0.00001; heterogeneity Chi^2 ^= 7.38, df = 3, *P *= 0.06, I^2^ = 59%] (Figure 3).

#### VAS leg pain scores at 1 day

**Figure 4:**
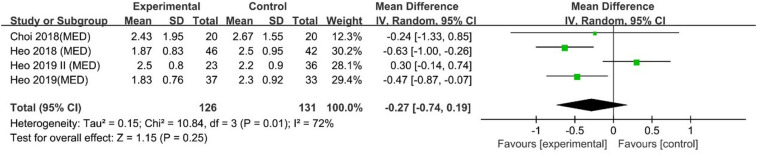
Forrest plot of vas leg pain 1-day UBE vs MED.

Heo 2019 and Heo 2018 reported that UBE was superior to MED for 1-day VAS leg pain scores. However, Choi 2018 and Heo 2019 II reported no difference. A pooled analysis indicated that UBE was similar to MED regarding 1-day VAS leg pain scores [MD: −0.27, 95% CI: (−0.74, 0.19), *P* = 0.25; heterogeneity Chi^2 ^= 10.84, df = 3, *P *= 0.01, I^2^ = 72%] (Figure 4).

#### Long-term VAS back pain scores

**Figure 5:**
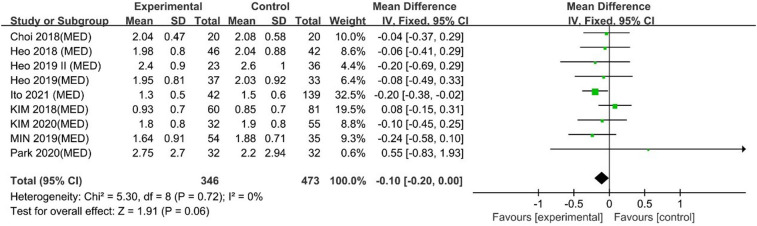
Forrest plot of long term vas back pain UBE vs MED.

**Figure 6:**
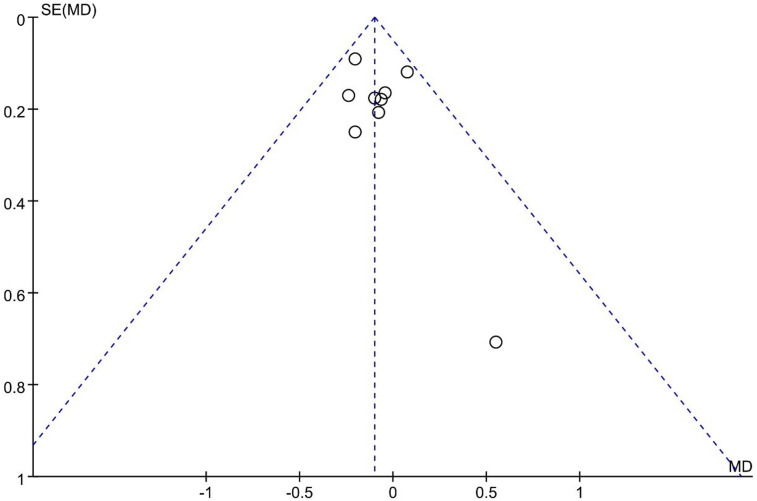
Publication bias of long term Vas back pain UBE vs MED.

Ito 2021 reported that UBE resulted in better long-term VAS back pain scores than MED, and 8 other studies reported similar efficacy. However, a meta-analysis indicated that UBE was similar to MED in long-term VAS leg pain scores [MD −0.10, 95% CI: (−0.20, 0.00), *P* = 0.06; heterogeneity Chi^2 ^= 5.30, df = 8, *P *= 0.72, I^2^ = 0%] (Figure 5). The degree of publication bias was low (Figure 6).

#### Long-term VAS leg pain scores

**Figure 7:**
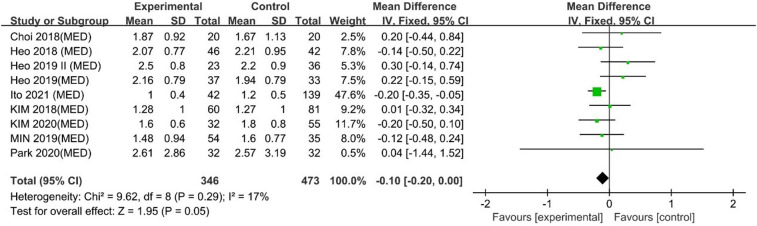
Forrest plot of long term vas leg pain UBE vs MED.

**Figure 8:**
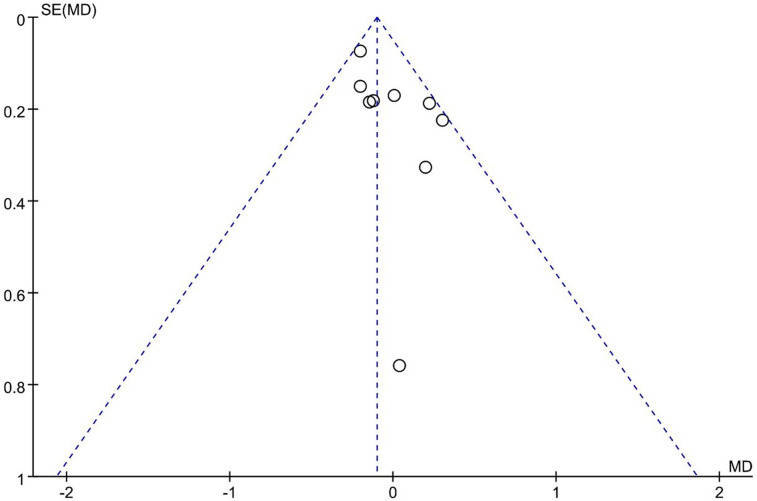
Publication bias of long term Vas leg pain UBE vs MED.

Ito 2021 reported that UBE outperformed MED in long-term VAS leg pain scores, and 8 other studies reported similar efficacy. However, the pooled analysis indicated that UBE was similar to MED in long-term VAS leg pain scores (MD: −0.10, 95% CI: (−0.20, 0.00), *P* = 0.05; heterogeneity Chi^2 ^= 9.62, df = 7, *P *= 0.29, I^2^ = 17%] (Figure 7). The degree of publication bias was low (Figure 8).

#### ODI

**Figure 9:**
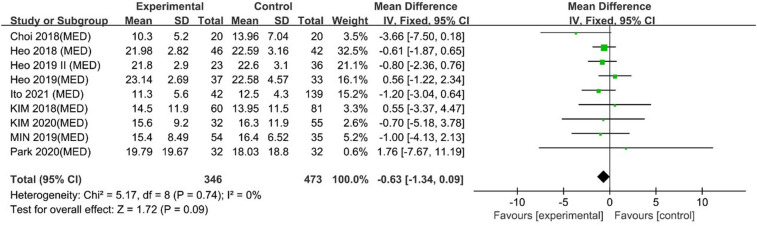
Forrest plot of ODI UBE vs MED.

**Figure 10:**
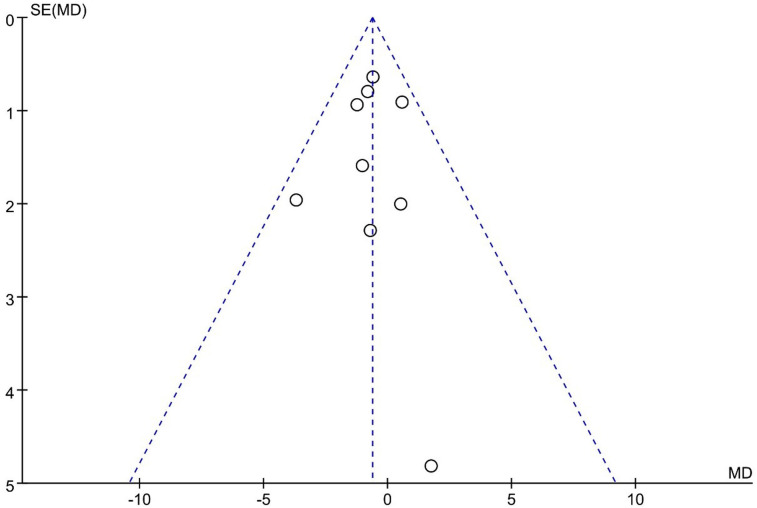
Publication bias of ODI UBE vs MED.

UBE was similar to MED regarding ODI scores [MD: −0.63, 95% CI: (−1.34, 0.09), *P* = 0.09; heterogeneity Chi^2 ^= 5.17, df = 8, *P *= 0.74, I^2^ = 0%] (Figure 9). The degree of publication bias was low (Figure 10).

#### Complications

**Figure 11:**
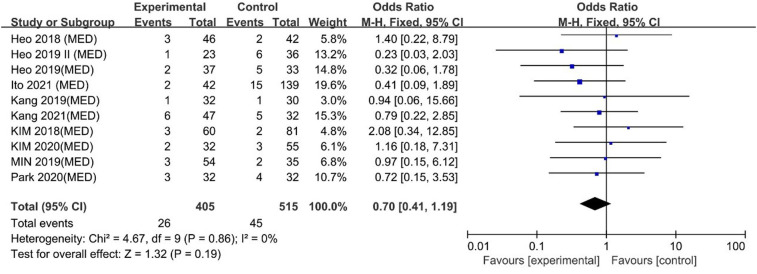
Forrest plot of complications UBE vs MED.

**Figure 12:**
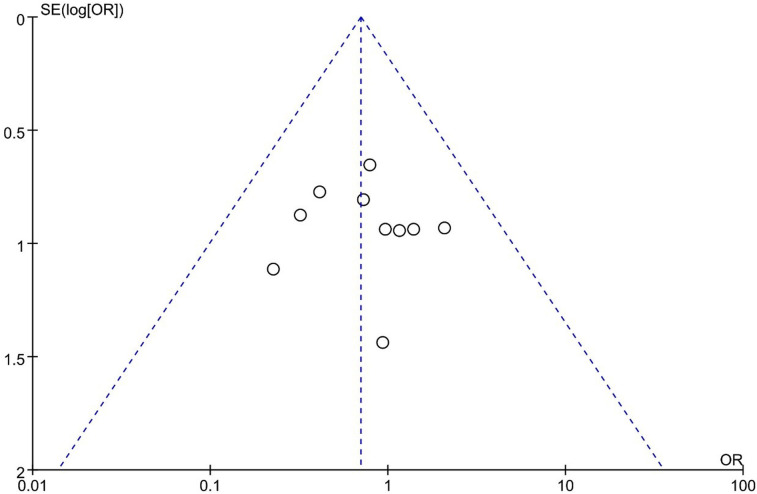
Publication bias of complications UBE vs MED.

No difference was found between UBE and MED regarding complications [OR: 0.70, 95% CI: (0.41, 1.19), *P* = 0.19; heterogeneity Chi^2 ^= 4.67, df = 9, *P *= 0.86, I^2^ = 0%] (Figure 11). The funnel plot indicated low publication bias (Figure 12).

### 3.4 UBE vs. PELD

#### VAS back pain scores at 1 day

**Figure 13:**
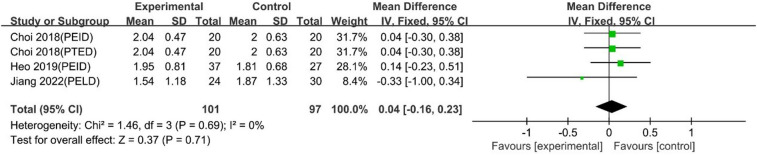
Forrest plot of vas back pain 1-day UBE vs PELD.

UBE was similar to PELD regarding 1-day VAS back pain scores [MD: 0.04, 95% CI: (−0.16, 0.23), *P* = 0.71; heterogeneity Chi^2 ^= 1.46, df = 3, *P *= 0.69, I^2^ = 0%] (Figure 13).

#### VAS leg pain scores at 1 day

**Figure 14:**
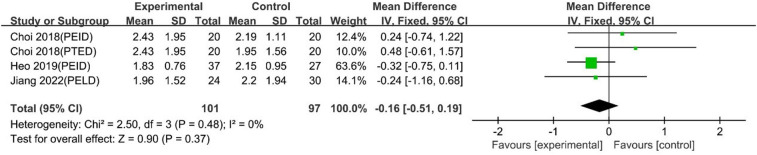
Forrest plot of vas leg pain 1-day UBE vs PELD.

UBE was similar to PELD regarding 1-day VAS leg pain scores [MD: −0.16, 95% CI: (−0.51, 0.19), *P* = 0.37; heterogeneity Chi^2 ^= 2.50, df = 3, *P *= 0.48, I^2^ = 0%] (Figure 14).

#### Long-term VAS back pain scores

**Figure 15:**
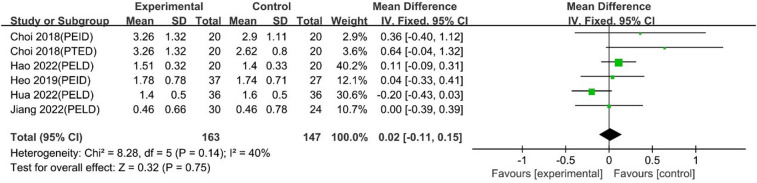
Forrest plot of long term vas back pain UBE vs PELD.

The pooled analysis, including 5 studies with 6 comparison groups, showed that UBE was similar to PELD in long-term VAS back pain scores [MD: 0.02, 95% CI: (−0.11, 0.15), *P* = 0.75; heterogeneity Chi^2 ^= 8.28, df = 5, *P *= 0.14, I^2^ = 40%] (Figure 15).

#### Long-term VAS leg pain scores

**Figure 16:**
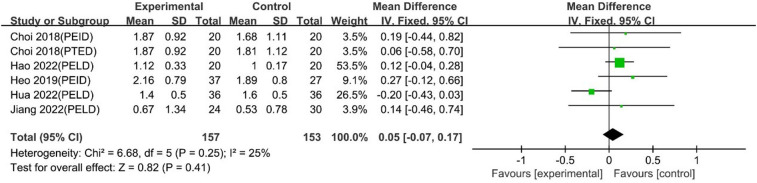
Forrest plot of long term vas leg pain UBE vs PELD.

The pooled analysis, including 5 studies with 6 comparison groups, showed that UBE was similar to PELD in long-term VAS leg pain scores [MD: 0.05, 95% CI: (−0.07, 0.17), *P* = 0.41; heterogeneity Chi^2 ^= 6.68, df = 5, *P* = 0.25, I^2^ = 25%] (Figure 16).

#### ODI scores

**Figure 17:**
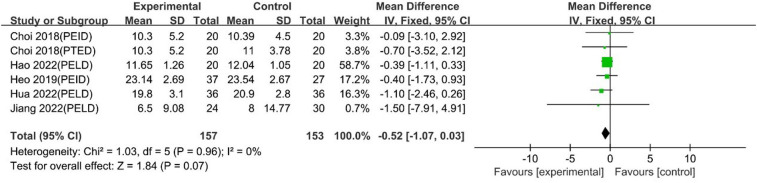
Forrest plot of ODI UBE vs PELD.

The pooled analysis, including 5 studies with 6 comparison groups, showed that UBE was similar to PELD regarding ODI scores [MD: −0.52, 95% CI: (−1.07, 0.03), *P* = 0.07; heterogeneity Chi^2 ^= 1.03, df = 5, *P *= 0.96 I^2^ = 0%] (Figure 17).

#### Complications

**Figure 18:**
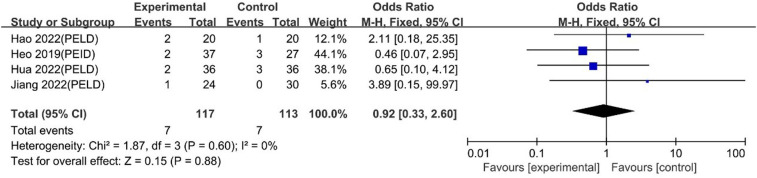
Forrest plot of complications UBE vs PELD.

Choi 2018 reported no complications. Heo 2019, Hao 2022, Hua 2022 and Jiang 2022 reported no difference in complications, and the meta-analysis indicated similar degrees of safety between UBE and PELD [OR: 0.92, 95% CI: (0.33, 2.60), *P* = 0.88; heterogeneity Chi^2 ^= 1.87, df = 3, *P *= 0.60, I^2^ = 0%] (Figure 18).

### UBE vs. PLIF

Park 2019 compared UBE and PLIF and found no difference in long-term VAS back pain scores, long-term VAS leg pain scores, ODI scores or complications. More evidence is needed to compare UBE and PLIF.

## Discussion

The rapid development of minimally invasive surgery has resulted in novel techniques for treating spinal diseases. Minimally invasive surgery is defined by more than the small size of the skin incision. A procedure that addresses damage to vertebrae and muscles under a small incision cannot be considered minimally invasive. The aim of minimally invasive surgery is to achieve adequate decompression while minimizing surgery-related trauma and postoperative spinal instability.

UBE is a novel endoscopic procedure for treating spinal disorders. In UBE, two portals are created on the same side; one is for the optical instrument and irrigation system, and the other is for the surgical instrument used to perform decompression or discectomy. The clear surgical field allows safe and adequate decompression. Unlike PELD, UBE is procedurally and anatomically similar to the traditional surgical process. Experienced surgeons can combine their knowledge of classic spine anatomy with the skill of spine endoscopy. UBE utilizes the natural gap between muscles to avoid unnecessary damage to the spine and associated structures. The advantages of UBE include less damage to the paraspinal musculature, smaller wounds, and better surgical vision. (23, 24) The major challenges in UBE are establishing suitable saline irrigation, minimizing complications, and properly localizing the surgical region (25, 26).

PELD and MED, two commonly applied forms of minimally invasive spinal surgery, have been widely reported to have satisfactory effectiveness and safety. However, conventional open surgery remains the most common treatment for spinal canal stenosis and disc herniation. UBE, as a novel form of minimally invasive spine surgery, requires comparison to mature spine surgery to verify its efficacy and safety.

Pain is the most common chief complaint leading to spine surgery. Therefore, effective pain relief is the goal of most spine surgeries. (27) In most common spine diseases, pain results from compression of the spinal cord or nerve roots. Adequate nerve decompression is the key to achieving a good clinical prognosis. All included studies reported a rapid decrease in pain immediately after UBE surgery, with improvement in VAS scores. UBE also has satisfactory long-term efficacy compared with the perioperative baseline.

In the long-term pain evaluation, no difference was found between UBE and other forms of spinal surgery, such as PELD, MED, and PLIF. UBE, as well as other surgeries, can provide satisfactory pain improvement and quality of life. No difference was observed in long-term pain scores, indicating similar degrees of efficacy between UBE and other surgical procedures. The long-term effects depend on complete decompression around the compressed nerve roots or spinal cord to prevent recurrence. Thus, all the procedures provided sufficient decompression to achieve long-term efficacy.

Regarding ODI outcomes, the pooled analysis indicated no difference between UBE and MED or PELD. The ODI evaluation reflects quality of life after surgery. Pain and other symptoms caused by nerve compression are the major influential factors. In each group, long-term pain improved compared with the perioperative baseline, and the meta-analysis indicated no difference between groups. The similar efficacy of these procedures for pain relief can explain the similar quality of life between groups after surgery.

No difference was found between UBE and PELD or MED regarding instant leg pain relief. Because long-term leg pain was similar between the groups, all procedures provided sufficient relief from nerve compression. Radiating leg pain originates from nerve root compression. Therefore, UBE results in similar leg scores to PELD and MED because of adequate decompression.

All three groups achieved an immediate reduction in back pain compared with the perioperative baseline. For intergroup comparisons, UBE was as effective as PELD and superior to MED. Patients who had undergone UBE had lower VAS back pain scores than those who had undergone MED. Pain scores were not significantly different between the UBE and PELD groups. Long-term back pain analysis indicated that the efficacy of UBE was similar to that of PELD and MED, indicating an adequate degree of nerve decompression in all three groups. Therefore, the difference in VAS back pain scores at 1 day can be attributed to iatrogenic posterior musculoligamentous damage by surgical equipment. The muscle-stripping technique is applied in MED, and the muscle-splitting technique with sequential dilators and a blunt obturator is applied in PELD. UBE combines the muscle splitting and muscle stripping techniques. Splitting, stripping, dissection, retraction and cauterization of ligaments and muscles during surgery lead to iatrogenic pain postoperatively. Ito 2021 reported less bone resection in the UBE group than in the MED group. Therefore, regarding surgical damage, UBE is similar to PELD and more favorable than MED. Park 2019 did not compare short-term pain scores between UBE and PLIF. In theory, UBE is less invasive than PLIF, but more evidence is needed.

Because UBE is a novel procedure, safety should not be ignored. A pooled analysis reported no difference between UBE and MED or PELD regarding complications. Park 2019 reported similar results between UBE and PLIF. However, more evidence is needed to evaluate the comparative safety of UBE and PLIF. In addition to complications, surgeons and patients cannot ignore cumulative radiation exposure. Among PELD, UBE, and MED, Meter reported that PELD entailed the most radiation exposure, followed by UBE and MED. (28) However, high-quality RCTs comparing radiation exposure procedures are lacking.

## Strengths and limitations

This study is the first comprehensive summary and meta-analysis of published studies comparing UBE to other forms of spine surgery regarding efficacy and safety. However, several limitations exist as follows: (1) Only papers published in English were included; therefore, studies published in other languages might have been neglected. (2) Because of the limited number of RCTs, both prospective and retrospective studies were included. In contrast to RCTs, prospective and case–control studies do not constitute high-grade evidence according to the Cochrane criteria. (3) The follow-up schedules of the included studies were not always adequate. Some studies failed to evaluate 1-day VAS scores. Additionally, the length of follow-up varied from one month to one year after surgery; a follow-up period of one month (Choi 2018) is not sufficient to evaluate the long-term effects. Hao 2022 reported a 6-month follow-up. (4) Traditional open surgeries are important parts of spine surgery. However, only one study in Park 2019 reports a comparison between UBE and PLIF, and more clinical trials are needed for further analysis. Additionally, comparison between UBE and traditional open surgery, such as TILF or OLIF, is lacking. This study lacks analysis between UBE and traditional open surgery.

## Future studies

More multicenter RCTs comparing UBE to other forms of spinal surgery, including traditional open surgery and spinal endoscopy, are needed to evaluate the efficacy and safety of UBE.

Spinal endoscopy is increasingly common worldwide. Guidelines should identify major indications and counterindications for UBE and other spinal endoscopic procedures. Minimally invasive surgery aims to minimize damage while maximizing efficacy and safety rather than minimizing the size of the skin incision.

## Conclusions

Unilateral biportal endoscopy is superior to MED in the relief of back pain at 1 day postoperatively. One-day leg pain relief, long-term effects and safety are similar between UBE and MED. UBE and PELD are similar regarding 1-day pain relief, long-term effects and safety. More evidence is needed to evaluate the efficacy and safety of UBE compared to PLIF.

## Data Availability

The original contributions presented in the study are included in the article/Suplementary Material, further inquiries can be directed to the corresponding author/s.
